# Early Onset Age Increased the Risk of Diabetic Retinopathy in Type 2 Diabetes Patients with Duration of 10–20 Years and HbA1C ≥7%: A Hospital-Based Case-Control Study

**DOI:** 10.1155/2021/5539654

**Published:** 2021-06-10

**Authors:** Jing Yuan, Lin Zhang, Pu Jia, Zhong Xin, Jin-Kui Yang

**Affiliations:** ^1^Department of Endocrinology, Beijing Tongren Hospital, Capital Medical University, 1 Dong Jiao Min Xiang, Beijing 100730, China; ^2^Department of Orthopaedics, Beijing Friendship Hospital, Capital Medical University, Beijing 100050, China

## Abstract

**Background:**

The relationship between onset age of diabetes and diabetic retinopathy (DR) is controversy and not concluded. Therefore, this hospital-based case-control study aimed to investigate the influence of diabetes onset age on the development of DR in patients with type 2 diabetes (T2D), independent of diabetic duration and HbA1c levels.

**Methods:**

A sample of 780 T2D patients with diabetic duration of 10–20 years and glycated hemoglobin (HbA1c) ≥7% were enrolled in the study. 338 T2D patients with onset age ≤45 years were further selected as cases (early onset) and 79 with onset age ≥ 55 years were chosen as controls (elderly onset). International Clinical Diabetic Retinopathy Disease Severity Scale was applied to estimate the severity of DR.

**Results:**

The prevalence of DR and proliferative diabetic retinopathy (PDR) was notably increased in the early onset group. When stratified by duration of diabetes, the impact of younger age on the risk of DR turned to be greatest in patients with diabetic duration ≥15 years (OR = 5.202, 95% CI 2.625–10.310). In groups stratified by HbA1c, the risk of DR was highest in patients with younger onset age and HbA1c ≥ 9% (OR = 3.889, 95% CI 1.852–8.167). Compared with the elderly onset group, the risk of DR (OR = 1.776, 95% CI = 1.326–2.380, *p* < 0.001) and PDR (OR = 1.605, 95% CI = 1.106–2.329, *p* = 0.013) in younger diagnosed patients was increased after multivariable adjustment.

**Conclusions:**

Age of onset was an independent risk factor for developing DR and PDR. This suggests that it is urgent to closely monitor and treat the metabolic disorders in younger T2D patients to delay the occurrence and progression of DR.

## 1. Introduction

Diabetic retinopathy (DR) is a prevalent and important microvascular complication of diabetes and widely considered as a major risk factor for vison loss and vison impairment in working-aged adults [1, 2], which could lead to decreased quality of life and increased financial burden of society. A Chinese epidemiological survey with 13473 diabetic patients demonstrated that the estimated prevalence of any DR and sight-threatening DR was 34.08% and 13.13%, respectively [3]. Previous studies [4–7] have already offered insights that hyperglycemia and long duration of diabetes are well-known risk factors in the development and progression of DR. Other risk factors, such as hypertension [8] and high lipid levels [9], are also reported to be positively related with DR. Although accumulative studies have been conducted in order to better understand the pathogenic features of DR during the last decades, the risk factors of DR are still not equivocally elucidated.

There is a growing recognition that onset age of diabetes might be an underlying influencing factor to the risk of DR. Some studies reported that patients with early onset diabetes and with longer diabetic duration [10] or higher HbA1c [11] had an increased prevalence of DR. And another study indicated that type 2 diabetes (T2D) patients with onset age of 31–45 years, not ≤30 years, had the highest susceptibility to DR [12]. Meanwhile, a study suggested that age at diabetes onset, diabetes could not predict the risk of DR at 17 years of diabetic duration [13]. Therefore, the issue is controversy and not concluded. To the best of our knowledge, a case-control study is not applied to date to minimize the influence of diabetic duration and glycemic control.

This study aimed to investigate whether onset age of diabetes was an independent risk factor to DR under a certain range of diabetic duration and HbA1c levels.

## 2. Methods

### 2.1. Study Population

**Figure 1:**
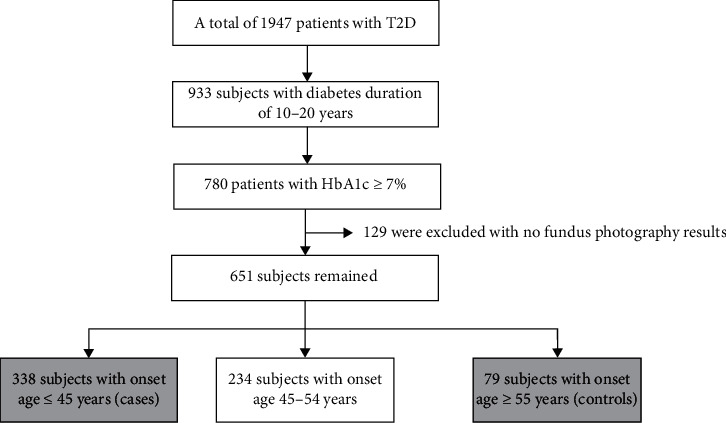
Flow chart of the study.

This was a hospital-based case-control study. We screened a total of 1947 T2D patients aged ≥18 years who were admitted to the Department of Endocrinology, Beijing Tongren Hospital, Capital Medical University, from January 2015 and May 2019, in Beijing, China. Based on inclusion criteria, a sample of 780 participants with diabetic duration of 10–20 years and glycated hemoglobin (HbA1c) ≥7% were selected in the study. And 129 subjects with no results of fundus photography were excluded from the study. After that, 338 T2D patients with onset age ≤45 years were selected as cases (early onset) and 79 with onset age ≥ 55 years were chosen as controls (elderly onset). The flowchart of the study is shown in Figure 1.

### 2.2. Anthropometric and Laboratory Measurements

Demographic details of patients were recorded, including age, sex, age of diagnosis, duration of diabetes, and smoking status. Smoking status was classified as never, former, or current. Antidiabetic treatment was also recorded. Body mass index (BMI) was calculated as the weight (kg) divided by the square of height (m^2^). Blood pressure (BP) was measured when the patients maintained a supine position and had a 10 minutes rest. Blood samples were taken at an overnight fasting status. Fasting blood glucose (FBG), serum creatinine (Scr), uric acid (UA), alanine aminotransferase (ALT), aspartate aminotransferase (AST), total cholesterol (TC), triglycerides (TG), and low-density lipoprotein (LDL) cholesterol concentrations were measured by an automated biochemical analyzer (Beckman company, US). HbA1c was assessed by high-performance liquid chromatography (VARIANT, BioRad Lab., Hercules, CA, US). Fasting blood C-peptide was measured by electrochemiluminescence (Roche, Germany).

### 2.3. Diabetic Retinopathy Assessment

As we described in the previous study, DR was determined using a TopconTRC-NW7SF fundus camera (Topcon, Tokyo, Japan) ophthalmic digital imaging system (2 eyes × 7 fields). These photographs were examined independently by two rigorously trained certified retinal photography graders while following quality assurance protocols. International Clinical Diabetic Retinopathy Disease Severity Scale [14] was applied to estimate the severity of DR as the following categories: nondiabetic retinopathy (NDR), nonproliferative diabetic retinopathy (NPDR), and proliferative diabetic retinopathy (PDR).

### 2.4. Statistical Analysis

Continuous variables were expressed as mean ± standard deviation $\left(\bar{\chi }\pm s\right)$, and the comparison between 2 groups was made using a *t*-test. Categorical measures were expressed as percentage, and a chi-squared test was used for analysis. Binary logistic regression analysis was used to calculate crude and adjusted odds ratio (OR) to estimate the influencing factors of DR in hospitalized T2D patients. Statistical analyses were performed using SPSS ver. 17.0 software (SPSS Inc., Chicago, IL, US). A *p* value < 0.05 was considered to be a statistically significant difference.

## 3. Results

### 3.1. Characteristics of T2D Patients

The demographic and biochemical characteristics of the early onset and elderly onset group are given in Table 1. Compared with the elderly onset group, the prevalence of DR (28.2% for controls vs. 54.7% for cases, *p* < 0.001) and PDR (14.1% for controls vs. 25.5% for cases, *p* < 0.05) was notably increased in the early onset group. The early onset group tended to have longer duration of diabetes, lower serum creatinine levels, less favorable lipid metabolism, and more current smokers (*p* < 0.001). More patients in the early onset group were treated by insulin (*p* < 0.001). There were no significant differences in sex, BMI, HbA1c, C-peptide, FBG, and UA (*p* > 0.05).

### 3.2. Effects of Onset Age of Diabetes on Risk of DR Stratified by Duration of Diabetes or HbA1c

**Figure 2:**
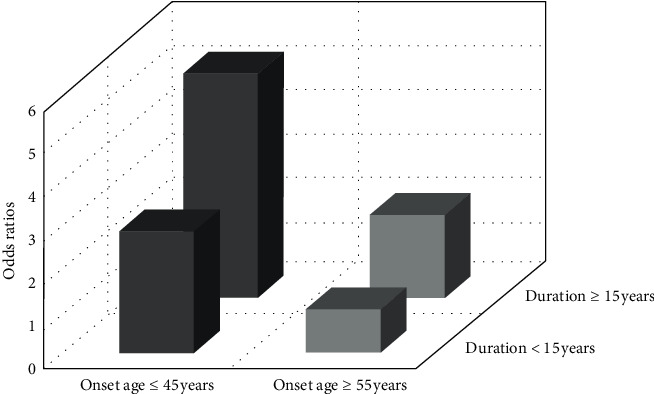
Odds ratios of DR by onset age of T2D and category of diabetic duration.

Patients were divided into two groups according to duration of diabetes (<15 years and ≥15 years) and HbA1c (7–9% and ≥9%), respectively. As given in Table 2 and Figure 2, in groups stratified by duration of diabetes, the risk of DR in patients with younger age of diagnosis and diabetic duration ≥15 years was greatest (OR = 5.202, 95% CI 2.625–10.310), with approximate 2.7 times increase in the OR of retinopathy compared with patients of similar diabetic duration but diagnosed at ≥55 years (OR = 1.934, 95% CI 0.666–5.619). Similarly, in groups stratified by HbA1c, the impact of early onset age on the risk of DR turned to be highest in T2D patients with HbA1c ≥ 9% (OR = 3.889, 95% CI 1.852–8.167). The association between onset age and PDR stratified by diabetic duration or HbA1c was still significant but attenuated.

### 3.3. Association between Onset Age of Diabetes and the Risk of DR

The association between onset age of diabetes and the risk of DR was analyzed in 4 models (Table 3). Model 1 was a crude model. Model 2 was adjusted for sex and BMI. Model 3 was further adjusted for HbA1c and diabetic duration. Model 4 was additionally adjusted for serum creatinine, TG, and hypertension. Early onset age of diabetes was associated with higher risk of DR (OR = 1.749, 95% CI = 1.338–2.287, *p* < 0.001) in univariate analysis (model 1). After adjustment for model 2, model 3, and model 4, the association remained statistical significant. Compared with the elderly onset group, the risk of PDR in younger diagnosed patients was also increased (OR = 1.452, 95% CI = 1.003–2.0437, *p* = 0.032) for model 1, and the age of onset was an independent risk factor for developing PDR after multivariable adjustment.

## 4. Discussion

In this hospital-based case-control study with T2D patients of diabetic duration 10–20 years and HbA1c ≥ 7%, we demonstrated that the prevalence of DR and PDR was significantly higher in early onset T2D patients (≤45 years) than in elderly onset T2D patients (≥55 years). When further stratified by duration of diabetes and glycemic control, we found that the influence of early onset age on the risk of DR was highest in T2D patients with diabetic duration ≥15 years (OR 5.202, 95% CI 2.625–10.310) and HbA1c ≥ 9% (OR 3.889, 95% CI 1.852–8.167). Age of onset was an independent risk factor for developing DR and PDR after multivariable adjustment with traditional risk factors of DR.

Previous studies reported that early onset diabetes was more aggressive [15–17] and may be related to increased occurrence of diabetic microvascular [18]. Wong et al. [10] analyzed data from 624 T2D patients with duration of 20–30 years (group A) and 852 T2D patients with duration of 10–20 years (group B). The results suggested that early onset age (<45 years) was an independent risk factor for DR after adjusted for traditional risk factors; however, disease duration, known as an important risk factor to DR, was not entered into the statistical model. Furthermore, the findings of the ADVANCE trial [19] reported a remarkable interaction between age or age at diagnosis and diabetes duration on the risk of microvascular events, and the highest risk of microvascular events was found in groups with the longest diabetes duration and the youngest age. In addition, a Chinese study with 29442 T2D patients of 77 tertiary hospitals indicated that the increased risk of DR in the early onset group was attributable to prolonged diabetes duration [20]. Therefore, it is essential to contain “diabetes duration” in the statistical model to evaluate the independent effect of early onset age on the development and progression of DR. In our study, we found that early onset age was associated with increased risk of DR, independent of diabetes duration, hyperglycemia, and other traditional risk factors of DR.

The relationship between age at onset and the risk of DR may not be limited to T2D. The FinnDiane Study [21] reported that compared to T1D patients with age at onset of 0–5 years and 15–40 years, group of 5–14 years had the highest risk of PDR, with HbA1c, blood pressure, sex, and BMI as covariates. A retrospective cohort study [22] enrolled 153 T1D patients with onset of diabetes < 18 years and suggested that older onset age was associated with development of DR 10 years after diagnosis. Some studies [23] indicated that puberty or delayed menarche could accelerate the progress of microvascular complications in T1D patients. Given the controversial results in T1D patients and underlying different mechanisms of DR between the two types of diabetes, it is inappropriate to mix T1D and T2D patients together for data analysis, as some previous studies conducted [24], which may not achieve accurate results. Therefore, the current study only enrolled T2D patients for analysis.

The potential mechanisms are still unclear. Several explanations may interpret the association between onset age and DR. First, more insulin could be secreted in *β*-cells of mature mice and humans than in young *β*-cells under the stimulation of high glucose concentrations [25–27], while some studies reported patients with better *β*-cell function had a reduced incidence of DR [28]. Second, the levels of angiogenin [29], vascular endothelial growth factor (VEGF) [30], and advanced glycation end products [31], which play a crucial role, in angiogenesis and in the development of DR, vary with age in diabetic patients. Third, the metabolic disorder may be more serious in early onset diabetes than in late-onset diabetes, which could lead to susceptibility to DR. Multiple factors such as genetic, socioeconomic, psychological, and behavioral factors [32] may involve in the pathogenesis.

There are several strengths and limitations of our study. One strength is that, to the best of our knowledge, it is the first case-control study to evaluate the association between onset age and DR. All patients selected in the study had long duration of diabetes (10–20 years) and high levels of blood glucose (HbA1c) ≥7%, which ensure the possibility to develop DR. Another strength is that the influence of diabetes onset age on the development of PDR was also investigated, which was previously conducted in only a few studies with T1D patients [21, 33]. PDR is the most severe form of DR and could seriously affect the vision. We found that the prevalence of PDR was increased, and odds ratio of PDR was 1.605 (95% CI: 1.106–2.329) in early onset diabetes. A limitation of the study was that as a hospitalized study, the T2D patients of our study may not be representative of the actual demographic profile of T2D patients in Beijing. Second, because of the nature of observation study, it was difficult to conceive the cause-effect relationship. Third, DR was measured only by fundus photography. Fundus fluorescence angiography and optical coherence tomography are needed in the future research.

## 5. Conclusions

In summary, our study shows a significant relationship between onset age and the risk of DR in T2D patients. This suggests that it is urgent to closely monitor and treat the metabolic disorders in younger T2D patients to delay the occurrence and progression of DR.

## Figures and Tables

**Table 1:** Demographic and clinical characteristics of type 2 diabetes patients.

	Onset age ≤45 years (*n* = 338)	Onset age ≥55 years (*n* = 79)	*p*
Age (years)	53.58 ± 6.65	74.27 ± 5.59	<0.001
Duration of diabetes (years)	15.29 ± 3.51	13.66 ± 3.57	<0.001
Male, *n* (%)	210 (62.1%)	45 (57.0%)	0.396
Smoking status, *n* (%)			
Never	178 (52.7%)	64 (81.0%)	<0.001
Former	44 (13.0%)	10 (12.7%)	
Current	116 (34.3%)	5 (6.3%)	
BMI (kg/m^2^)	25.87 ± 3.34	25.82 ± 3.41	0.908
HbA1c (%)	9.19 ± 1.49	9.05 ± 1.58	0.460
Fasting blood C-peptide (ng/ml)	1.74 ± 1.15	2.07 ± 1.31	0.245
FBG (mmol/l)	8.28 ± 2.76	8.37 ± 2.46	0.807
SBP (mmHg)	133.78 ± 17.14	135.61 ± 19.10	0.485
DBP (mmHg)	78.39 ± 10.68	74.93 ± 12.39	0.037
Scr (mmol/l)	69.17 ± 22.44	80.15 ± 26.27	<0.001
UA (mmol/l)	345.05 ± 82.48	333.87 ± 90.75	0.291
ALT (U/l)	22.82 ± 15.56	19.42 ± 12.78	0.162
AST (U/l)	22.28 ± 13.25	21.44 ± 9.64	0.154
TC (mmol/l)	4.59 ± 1.17	3.96 ± 0.93	<0.001
TG (mmol/l)	2.21 ± 1.83	1.59 ± 0.97	<0.001
LDL (mmol/l)	2.71 ± 0.97	2.29 ± 0.79	<0.001
Antidiabetic treatment, *n* (%)			
Oral hypoglycemia agent	318 (94.1%)	73 (92.4%)	0.579
Insulin	304 (89.9%)	56 (70.9%)	<0.001
GLP-1RA	20 (5.9%)	2 (2.5%)	0.226
DR (%)	183 (54.1%)	22 (27.8%)	<0.001
PDR (%)	86 (25.4%)	11 (13.9%)	<0.001

BMI, body mass index; HbA1c, glycated hemoglobin; FBG, fasting blood glucose; SBP, systolic blood pressure; DBP, diastolic blood pressure; Scr, serum creatinine; UA, uric acid; ALT, alanine aminotransferase; AST, aspartate aminotransferase; TC, total cholesterol; TG, triglycerides; LDL, low-density lipoprotein; GLP-1RA, GLP-1 receptor agonist; DR, diabetic retinopathy; PDR, proliferative diabetic retinopathy.

**Table 2:** Odds ratios of DR and PDR by onset age of T2D and category of diabetic duration or HbA1c.

	DR		PDR	
	Early onset	Elderly onset	Early onset	Elderly onset
Diabetes duration				
<15 years	2.819 (1.446–5.495)	1 (ref)	3.039 (1.140–8.102)	1 (ref)
≥15 years	5.202 (2.625–10.310)	1.934 (0.666–5.619)	4.376 (1.642–11.666)	4.240 (1.135–15.840)
HbA1c (%)				
7–9%	1.914 (0.910–4.026)	1 (ref)	1.797 (0.702–4.598)	1 (ref)
≥9%	3.889 (1.852–8.167)	0.805 (0.300–2.159)	2.065 (0.815–5.232)	0.833 (0.232–2.993)

DR, diabetic retinopathy; PDR, proliferative diabetic retinopathy; T2D, type 2 diabetes; HbA1c, glycated hemoglobin.

**Table 3:** The risk of DR and PDR according to onset age of T2D.

	DR		PDR	
	OR (95% CI)	*p*	OR (95% CI)	*p*
Model 1	1.749 (1.338–2.287)	<0.001	1.452 (1.033–2.043)	0.032
Model 2	1.756 (1.342–2.298)	<0.001	1.476 (1.046–2.081)	0.027
Model 3	1.677 (1.273–2.207)	<0.001	1.403 (0.990–1.986)	0.057
Model 4	1.776 (1.326–2.380)	<0.001	1.605 (1.106–2.329)	0.013

Model 1 was unadjusted. Model 2 was adjusted for sex and BMI. Model 3 was adjusted for sex, BMI, HbA1c, and diabetic duration. Model 4 was adjusted for sex, BMI, HbA1c, diabetic duration, serum creatinine, TG, and hypertension. DR, diabetic retinopathy; PDR, proliferative diabetic retinopathy; T2D, type 2 diabetes; OR, odds ratio; BMI, body mass index; HbA1c, glycated hemoglobin; TG, triglycerides.

## Data Availability

The data used to support the findings of this study are available from the corresponding author upon request.
